# Supplementary material for: Legal education reform and medical litigation: Improved access but delayed justice in plastic surgery malpractice cases in South Korea
Source: PLoS One. 2025 May 29;20(5):e0323059. doi: 10.1371/journal.pone.0323059 (PMC12121745; doi:10.1371/journal.pone.0323059)
Supplement: Attachment — Submitted filename: Response to reviewers.docx [file pone.0323059.s003.docx]

**Response to reviews**

**Reviewer #1: General comments:**
Based on focus of the paper, the paper aligns with PLOS ONE's scope, offering an empirical analysis of legal education reform and its unintended consequences on malpractice litigation in South Korea. The research provides valuable insights, is well-structured, and uses appropriate statistical methods. However, the paper needs improvements in contextual analysis, causality interpretation, and broader applicability of findings before it becomes ready for publication.

Comments to be address by the author before acceptance:

**1. The abstract lacks details on causative factors and practical implications. Kindly revise the abstract to include more actionable insights for policymakers and practitioners.**

- Thank you for highlighting the need for a more detailed abstract. In response, we have revised the abstract to explicitly include causative factors, such as systemic inefficiencies and increased public awareness, as key drivers of the observed litigation trends. Additionally, we have integrated actionable insights for policymakers and practitioners, such as expanding judicial capacity and implementing alternative dispute resolution mechanisms.

**2. The statistical methods (Mann-Whitney U test, Fisher’s Exact Test) are appropriate. However, the manuscript should clearly explain the selection of statistical tests for each metric (e.g., why Monte Carlo simulations were needed for specific Fisher's tests). So please provide additional justification for the use of non-parametric tests for damages and liability attribution data?**

- Thank you for your thoughtful feedback on the statistical methods. In response, we have revised the manuscript to include a detailed explanation of the selection of statistical tests for each metric. Specifically:
- Use of Non-Parametric Tests: Non-parametric tests, such as the Mann-Whitney U test and Fisher’s Exact Test, were selected due to the non-normal distribution of the damages and liability attribution data. This was confirmed through preliminary normality testing, which showed skewness and kurtosis inconsistent with normality assumptions. Non-parametric methods provided a robust approach to analyze this data without violating statistical assumptions.
- Monte Carlo Simulations: The manuscript now clarifies that Monte Carlo simulations were employed to improve the accuracy of p-value estimations in Fisher’s Exact Test, especially for small sample sizes in specific contingency tables. This technique ensures reliability in hypothesis testing when traditional exact methods encounter computational constraints.

**3. While the data is detailed, adding more visual elements (e.g., trend graphs, heat maps) would make trends easier to interpret. Could you include graphical representations to highlight trends in litigation duration and geographical case distribution?**

- Thank you for your suggestion to enhance the manuscript with visual elements to improve data interpretation. In response, we have added the following graphical representations:
- Figure 1. Litigation Duration Trends & Figure 2. Geographical Distribution of Cases

**4. The study excludes cases from 2013–2017. While justified, this exclusion could create a potential gap in understanding the immediate impacts of the reform. Please explain how do you ensure that excluding data from this transitional period does not introduce bias? Would including some of these cases provide additional insights?**

- Thank you for raising this important point. In the revised manuscript, we have thoroughly elaborated on the rationale behind excluding cases from the transitional period (2013–2017). This decision was made to highlight statistically meaningful contrasts between the pre-reform and post-reform periods.
- During the transitional phase, the number of law school graduates entering the workforce was relatively low, which likely diluted the reform's impact on litigation patterns. Moreover, legal professionals in this early stage might not have fully developed their individual legal philosophies or practices, creating inconsistencies that could confound the analysis.
- While this approach may introduce some degree of selection bias, we have acknowledged this limitation in the revised Discussion section. Additionally, we emphasized that future studies incorporating this transitional period could provide further insights. Thank you for your valuable feedback, which helped refine the methodological clarity of the manuscript.

**5. The exclusion of small claims court decisions might omit a significant subset of medical disputes. Could the exclusion of small claims cases significantly affect the representativeness of your findings?**

- We appreciate the reviewer's valuable comment regarding the exclusion of small claims court decisions and their potential impact on the representativeness of our findings. While we recognize the importance of small claims cases in offering a more complete picture of medical disputes, including these cases in our analysis posed significant practical challenges. In South Korea, small claims cases are typically adjudicated by a single judge and, due to the heavy caseload and time constraints faced by judges, detailed written judgments are rarely produced for these cases. This observation is consistent with insights shared by practicing judges, who describe the overwhelming workload associated with small claims adjudication.
- As a result, the lack of accessible written judgments from small claims courts makes it difficult to systematically include these cases in our study. While we believe that the exclusion of small claims cases does not substantially alter the main findings of our study due to their relatively limited complexity and scope, we acknowledge that this limitation could restrict the comprehensiveness of our analysis.
- We hope the reviewer understands the practical limitations we faced and the efforts we made to ensure the quality and integrity of this study within these constraints. While we recognize that further improvements are always possible, we believe this study offers meaningful insights given the realities of the available data and the broader judicial context.

**6. The increase in litigation duration is well-documented but requires more discussion on potential causes (e.g., systemic inefficiencies, strategic legal approaches). So expand on why litigation durations have increased so significantly despite the influx of legal professionals?**

- We appreciate the reviewer's comment highlighting the need for further discussion on the potential causes behind the significant increase in litigation duration. In response, we have expanded this section in the manuscript to address the systemic inefficiencies and strategic legal approaches that contribute to prolonged case durations.

1. First, the increased influx of legal professionals following the legal education reform has not been matched by a proportional expansion of judicial infrastructure. The existing number of judges and court facilities remains insufficient to handle the growing caseload, leading to procedural delays.
2. Second, the shift in the legal training system from practical apprenticeship to academically focused law schools has impacted the practical readiness of new legal professionals. This has likely introduced inefficiencies in managing complex medical litigation, including procedural complexities and prolonged case preparation times.
3. Lastly, strategic legal approaches, such as repeated motions, extensive discovery phases, and appeals, have become more prevalent with the increased accessibility of legal representation. These strategies, often employed to strengthen a party's position, contribute to prolonged litigation timelines.

- We hope these revisions clarify and address the reviewer's concerns.

**7. The findings focus on South Korea but lack direct comparisons to similar legal reforms globally. Need comparison of South Korea’s litigation trends to those in other countries with similar legal reforms (e.g., Japan and US) to enhance the paper’s international relevance.**

- We appreciate the reviewer's suggestion to enhance the paper's international relevance by including comparisons to similar legal reforms in other countries. In response, we have expanded the discussion section to incorporate comparisons with Japan, China, and Germany, which have also undergone notable legal or judicial reforms.
- In Japan, the 2004 legal education reform introduced graduate-level law schools, similar to South Korea's system. However, Japan retained its Legal Training and Research Institute, which requires one year of practical training before legal practice. This system emphasizes hands-on experience, potentially enhancing the practical readiness of new legal professionals. While comprehensive data on litigation durations specific to medical malpractice are limited, Japan’s use of court-mediated settlements and structured judicial processes may contribute to relatively efficient handling of complex cases, including medical litigation.
- China’s Smart Court initiative, launched in 2017, leverages AI and blockchain technologies to enhance judicial efficiency. These innovations have significantly reduced judges’ workloads, streamlined case management, and shortened case durations. While South Korea has yet to adopt such technologies on a large scale, China’s experience highlights the potential for technology-driven solutions to address procedural delays in litigation.
- Germany’s approach focuses on alternative dispute resolution mechanisms, such as expert review panels (Gutachterkommissionen) and mediation offices (Schlichtungsstellen). These systems resolve over 90% of medical disputes outside traditional courts, significantly reducing litigation durations and costs. Germany’s experience underscores the importance of institutional support for pre-litigation resolution, which could be a valuable model for South Korea to consider.
- These international comparisons highlight how different approaches to legal education and judicial reform influence litigation trends. We hope these revisions adequately address the author's concerns.

**8. The policy suggestions (e.g., increasing judicial capacity, introducing alternative dispute mechanisms) are relevant but lack specifics or examples. Please provide case studies or examples of similar policy interventions in other jurisdictions to support your recommendations.**

- We have added examples from other jurisdictions in the revised manuscript. For instance, Japan has established specialized medical litigation departments in major district courts to handle complex cases efficiently, while Germany resolves over 90% of medical disputes through expert review panels and mediation offices, reducing court burdens. Additionally, China’s Smart Court initiative utilizes AI and blockchain technology to streamline judicial processes and improve efficiency. These examples highlight the practical feasibility of the proposed interventions and their potential applicability to South Korea.

**9. The role of South Korea’s cultural attitudes toward plastic surgery and litigation is briefly mentioned but not deeply analyzed. Please tell us how might societal perceptions of cosmetic surgery influence litigation trends?**

- The revised manuscript includes an expanded discussion on how societal perceptions of cosmetic surgery influence litigation trends in South Korea. The normalization of cosmetic procedures as tools for self-improvement and social success, driven by social media and digital beauty culture, has heightened patient expectations. When outcomes do not meet these expectations, disputes and litigation become more likely. Additionally, traditional Confucian values that discouraged legal action are gradually giving way to a more assertive approach, particularly among younger generations. These cultural and societal dynamics have been analyzed in greater detail in the revised text.

**10. The manuscript is well-written but includes technical terms (e.g., “Monte Carlo simulation”) that may be unfamiliar to a broader audience. Please simplify or explain technical terms to ensure accessibility for a wider readership.**

- The manuscript has been revised to include simplified explanations for technical terms such as “Monte Carlo simulation” and other statistical methods used in the analysis. Additional context has been provided to clarify their purpose and relevance, ensuring accessibility for a broader readership.

**Publishable with Major Revisions: The manuscript has potential, but it needs improvements in contextual analysis, comparative perspectives and clarity. If the author addresses the above comments, the paper could be a valuable contribution to PLOS ONE.**

- Thank you for your thoughtful evaluation and constructive feedback. As a medical professional primarily focused on writing medical research papers, receiving this type of review was both surprising and enlightening. Compared to the relatively concise manuscripts typical in plastic surgery, often limited to 2,500 words, it was clear that the expectations for this interdisciplinary work were quite different.
- This experience has been an invaluable opportunity to delve into social science and legal studies, broadening authors’ perspective and challenging us to write in a new academic domain. We hope this manuscript serves as a small step toward greater collaboration between medicine and the legal field, and that it contributes to a more integrated approach to scholarly writing in the future.
- Thank you for your time and effort in reviewing this paper.

**Reviewer #2: Comments / Observations on Manuscript titled:**

South Korea’s Legal Education Reform: More Lawyers, More Lawsuits, but Longer
Delays (Manuscript Number: PONE-D-24-54910)

The following are the comments/observations:

**1. The abstract should capture methodology of the study.**

- Thank you for pointing out this important aspect. The abstract has been revised to clearly capture the methodology of the study, including the use of retrospective analysis of publicly available civil court decisions and the comparative analysis of litigation patterns before and after the legal education reform.

**2. The introduction is not so explicitly elaborated to elicit various variables of the study.**

- Thank you for the constructive feedback. The introduction has been completely rewritten to explicitly elaborate on the key variables of the study, including litigation frequency, case duration, and adjudicated damages. Additionally, the revised introduction provides a clearer explanation of the study's scope, its relevance to South Korea's legal education reform, and the implications for the legal and medical communities.

**3. It is important to elucidate on the necessity of legal education reform in South-Korea, and explain the conceptualization of “more lawyers”, “more lawsuits” and “longer delays”.**

- Thank you for highlighting the importance of addressing the necessity of legal education reform in South Korea and clarifying the concepts of “more lawyers,” “more lawsuits,” and “longer delays.” These points have been incorporated into the revised Introduction, where the rationale behind the reform and its unintended consequences are more explicitly discussed. Specifically, the revised text elaborates on how the reform aimed to enhance legal expertise and expand access to justice, while also outlining the systemic challenges such as increased litigation frequency and prolonged case durations.

**4. The introduction should explain the rationale for the study, the gap to be filled and the novelty of the study.**

- This study addresses the gap in understanding how South Korea’s 2009 legal education reform impacted judicial efficiency and litigation behaviors. By analyzing civil court decisions in plastic and reconstructive surgery (PRSP), it investigates whether the reform led to "more lawsuits" and "longer delays." The research offers novel insights by focusing on a specialized legal domain and provides comparative lessons from other countries, contributing to global discussions on legal education reform.

**5. More literature review should be added to introduction since there is no subheading for literature review part. The following should be considered regarding the review of more studies:**
i. Explain a transition from a largely apprenticeship-based system to a more structured graduate-level education model in South-Korea.
ii. Concerns about quality which arose about inconsistencies in the quality of legal education and preparedness of graduates from the realities of legal practices should be elaborated in details.
iii. Standardized curriculum which reform was introduced covering core legal subjects should be explicated.
iv. Explain competitive entrance examination (LEET) which was introduced to select candidate for law school based on aptitude and academic achievement.
v. Explain current challenges and future direction.

- We sincerely appreciate the reviewer’s thoughtful suggestions to expand the literature review in the introduction. We agree that a detailed discussion of South Korea’s legal education reform, including its transition from an apprenticeship-based system to a graduate-level model, concerns about educational quality, standardized curricula, the LEET examination, and current challenges, would provide valuable context. However, as medical researchers adhering to the IMRaD structure, we have aimed to maintain a concise and focused introduction that aligns with the conventional format of scientific papers.
- The primary goal of this study is to highlight a relatively simple yet critical issue: the unintended consequences of the 2009 legal education reform, particularly the lack of follow-up measures to ensure judicial efficiency and access to justice. While we acknowledge the importance of broader societal, institutional, and legal discussions, delving deeply into all these aspects would exceed the scope of a research article and risk compromising its readability and focus. As the reviewer may note, the manuscript is already quite lengthy, and further expansion could detract from its clarity and impact.
- That said, we have made every effort to address the reviewer’s concerns within the constraints of the IMRaD format. We have added a brief but meaningful discussion of the reform’s background and its implications for judicial efficiency in the introduction, ensuring that the rationale for the study is well-grounded. We believe this approach strikes a balance between addressing the reviewer’s valuable feedback and maintaining the paper’s scientific rigor and accessibility.
- We hope the reviewer understands our position and agrees that the current manuscript, while concise, effectively communicates the study’s core message: that the legal education reform, while well-intentioned, has not fully achieved its original goals due to insufficient follow-up measures. We believe this message is both scientifically valid and socially relevant, and we are grateful for the opportunity to clarify our approach.

**6. Elaborate on how leveraging technology into legal education can enhance efficiency and learning outcomes in South-Korea as in the cases of UK and Singapore.**

- We appreciate the reviewer’s suggestion to elaborate on how leveraging technology in legal education can enhance efficiency and learning outcomes, particularly with reference to cases in the UK and Singapore. We agree that this is an important topic, and we have already addressed it in the revised Discussion section by incorporating examples of technological advancements in legal systems, such as China’s Smart Courts. These examples illustrate how technology can improve judicial efficiency and access to justice, which aligns closely with the reviewer’s point about the role of technology in legal education.
- While we have not explicitly discussed the UK and Singapore in this context, we believe that the inclusion of China’s Smart Courts provides a robust and relevant case study that sufficiently addresses the broader theme of technology’s impact on legal systems. We hope the reviewer finds this addition satisfactory, as it strengthens the manuscript’s discussion on the integration of technology into legal practices.
- Thank you for this constructive feedback, which has helped us enhance the manuscript’s depth and relevance.

**7. The researcher (s) should explore legal education reform from some selected countries such as: US, UK, Canada through legal education reform in South-Korea can learn from different lessons.**

- We thank the reviewer for the suggestion to explore legal education reforms in selected countries such as the US, UK, and Canada. We agree that comparative insights from these countries could provide valuable lessons for South Korea’s legal education system. In response to this feedback, we have already revised the Discussion section to include examples from Japan, China, and the United States, which offer relevant perspectives on balancing legal expertise with judicial efficiency and integrating technology into legal education.
- For instance, we have discussed Japan’s challenges in aligning legal education with practical needs, China’s advancements in leveraging technology through its Smart Courts, and the US’s emphasis on interdisciplinary legal training. While we have not explicitly addressed the UK and Canada, we believe that the inclusion of these cases provides a meaningful foundation for understanding the broader implications of legal education reform.
- We hope the reviewer finds these additions satisfactory, as they strengthen the manuscript’s comparative analysis and relevance to the global discourse on legal education reform.

**8. Regarding ethical considerations, the researcher (s) posited that: “This study was conducted in compliance with ethical standards for research”. Explain what you mean by ethical standards for research and justify with literature.**

- The study's ethical considerations align with international research standards and were conducted under explicit institutional oversight. The manuscript explicitly mentions Institutional Review Board (IRB) approval (File No. SEUMC NON2024-006), affirming compliance with ethical guidelines.
- Detailed Justification
  1. IRB Review and Exemption: The IRB reviewed the protocol, confirming that the study involves publicly available court data, exempting it from requiring direct participant consent. The use of non-sensitive, anonymized public records adheres to ethical norms as defined by guidelines like the Declaration of Helsinki.
  2. No Personal Data: Since no personal, sensitive, or identifiable data were collected, analyzed, or disclosed, the study avoids any risk to individuals' privacy or rights.
  3. Literature-Based Compliance: Ethical research in legal and medical studies emphasizes minimizing harm and maintaining transparency. This principle aligns with frameworks such as the Belmont Report, which underpins justice and respect in research involving human participants, even indirectly.

**9. Under “Data Analysis”, the researcher should write the full meaning of SPSS as shown the paper.**

- Thank you for your comment. The full meaning of SPSS, "Statistical Package for the Social Sciences," has been included in the revised manuscript under the "Data Analysis" section. This adjustment ensures clarity for readers who may not be familiar with the abbreviation.

**10. Tables1 to 4 should be incorporated into the appropriate places in the contents of the paper.**

- Thank you for your feedback. Tables 1 to 4 have been repositioned in the revised manuscript to appear immediately after the paragraphs where they are first referenced.

**11. The organization and arrangement of the paper should align with the standard of the journal.**

- The authors have reviewed the PLOS ONE guidelines and revised the manuscript to ensure alignment with the journal's standards.

**12. The results should be elaborated using various studies to justify the overall findings of the study.**

- The Results section has been revised to incorporate comparisons with existing studies and to contextualize the findings. Notably, extensive literature on medical malpractice litigation trends in countries such as Japan, China, and Germany has been reviewed and referenced where relevant. This comparison highlights similarities and differences in litigation frequency, case duration, and the impact of systemic factors on legal processes. By integrating these references, the revised manuscript provides a broader perspective and strengthens the discussion of the implications of South Korea's legal education reform.

**13. Recheck typographic, spelling and grammatical errors.**

- The manuscript has undergone multiple rounds of thorough proofreading and editing to ensure clarity and accuracy in language, grammar, and typographic consistency. Any remaining minor errors have been addressed in the revised version

**14. By incorporating all the foregoing, it will improve the quality of the paper in order to make legal education reforms to be tailored to South-Korea’s specific context, legal culture and improvement of societal needs.**

- All the aforementioned comments and suggestions have been carefully considered and addressed in the revised manuscript. These revisions have been made to enhance the paper's alignment with South Korea's specific legal context and societal needs, while also improving the overall quality and clarity of the study.

General answer:

We sincerely thank Reviewer #2 for their thoughtful and detailed feedback. As medical researchers who have spent our careers writing within the IMRaD framework, we must admit that some of the suggestions were quite surprising to us. In medical research, the introduction is typically concise, often limited to three paragraphs, and the entire manuscript is usually constrained to 2,500–3,000 words. This is a deeply ingrained convention in our field, and deviating from it feels almost unthinkable.

Given these constraints, we were initially taken aback by the request to expand the introduction with extensive background information, detailed discussions of legal education reforms (such as LEET and curriculum changes), and comparisons with other countries. While we recognize the importance of these points, incorporating them fully would likely push the manuscript far beyond the word limits we are accustomed to - perhaps even exceeding 10,000 words. This is far beyond the scope of a traditional medical research article.

That said, we have made every effort to address Reviewer #2’s concerns within the limits of the IMRaD format and our disciplinary background. For example, we have added a brief discussion of China’s Smart Courts and included comparative insights from Japan and Germany in the revised Discussion section. While these additions may not be as exhaustive as a legal scholar might expect, we believe they provide meaningful context and enhance the manuscript’s relevance to the broader discourse on legal education reform.

We hope Reviewer #2 understands our position and appreciates the challenges of balancing their valuable feedback with the conventions and constraints of medical research writing. While the manuscript may not be perfect from a legal studies perspective, we have done our best to incorporate the reviewer’s suggestions in a way that aligns with our expertise and the expectations of our primary audience. We are grateful for the opportunity to clarify our approach and hope the reviewer finds the revised manuscript satisfactory.

**Reviewer #3:**

The study provides valuable insights into the unintended consequences of South Korea’s legal education reform. It has significant value and potential but requires substantial improvements in methodology, analysis, and interpretation.

The methodology includes a retrospective analysis with clear pre- and post-reform time frames. The selection of publicly available court decisions aligns with the study's objectives. The criteria for case inclusion and exclusion are clearly defined, ensuring consistency and clarity in the dataset. However, broader legal and healthcare reforms during the study period may have influenced the findings, but these factors were not thoroughly explored. Furthermore, the pre-reform sample size is relatively small (n=23), limiting the statistical power of comparisons and potentially affecting the study's generalizability and robustness.

While the study identifies correlations, it does not establish a clear causal link between the reform and litigation trends. Without stronger causal evidence, the findings may not fully support the conclusions. The weaknesses in the methodology directly impact the reliability of the findings, necessitating significant revisions to address these gaps before the study’s claims can be confidently supported.

The discussion is moderately well-developed but requires substantial refinement to fully capitalize on the findings and contextualize them within broader academic and policy debates. While it effectively summarizes the results and offers high-level insights, the lack of depth and specificity undermines its impact. For instance, the discussion references some studies but does not engage deeply with the broader academic discourse on legal education reform or medical malpractice litigation. A more thorough comparison with international trends or prior studies could strengthen the discussion. Furthermore, integrating the findings with existing studies on legal education reform and medical litigation trends, particularly in South Korea and other countries with similar reforms, is recommended.

Additionally, deeper insights into the mechanisms driving the increase in litigation frequency and duration are needed. While these trends are attributed to broad factors such as greater public awareness and increased availability of legal services, the discussion lacks detailed explanations of these changes. Some conclusions overstate the findings, such as attributing judicial inefficiencies entirely to the reform without sufficient evidence. The recommendations are also broad and lack actionable specificity, such as how to implement alternative dispute resolution mechanisms or expand judicial capacity.

In short, the paper addresses an underexplored topic—the unintended consequences of legal education reform on medical malpractice litigation—which is valuable for both legal and medical disciplines, particularly given South Korea's unique context as a global leader in plastic surgery. The research provides a foundation that can be further developed.

However, the weaknesses in methodology, data analysis, discussion, and scope significantly undermine its reliability and generalizability. Additionally, the paper would benefit from language proofreading and editing. These issues require substantial revisions to strengthen the study’s contribution to academic and policy discourse.

**Answer:**

We sincerely thank you for your detailed and constructive feedback. Your insights have been instrumental in refining our manuscript and addressing its limitations. Below, we provide specific responses to each of your comments:

1. **Broader Legal and Healthcare Reforms**

We acknowledge the potential influence of broader legal and healthcare reforms during the study period. In response, we have added a dedicated subsection in the Discussion to address these factors and their potential impacts on litigation trends. While the primary focus remains on the legal education reform, this addition offers a more nuanced understanding of the findings within the broader socio-legal context.

2. **Sample Size and Generalizability**

We recognize the limitation of the small pre-reform sample size (n=23), which may affect the statistical power and generalizability of the study. This limitation has been explicitly discussed in the revised manuscript, along with the rationale for focusing on this dataset. We emphasize the need for future research with larger datasets to validate and extend our findings.

3. **Causal Links**

We agree that the retrospective design limits the ability to establish causality. This limitation has been further clarified in the revised Discussion. Additionally, we suggest directions for future research, such as longitudinal studies and qualitative analyses, to explore causal relationships more comprehensively.

4. **Discussion Depth and International Context**

The Discussion section has been significantly expanded to include comparisons with legal education reforms in Japan, China, and Germany. These international perspectives provide valuable context for interpreting our findings and highlight lessons that could inform South Korea’s ongoing reforms. Furthermore, the analysis of public awareness and increased access to legal services has been deepened to better explain the observed litigation trends.

5. **Mechanisms Behind Litigation Trends**

The revised manuscript includes a more detailed discussion of the mechanisms driving increased litigation frequency and duration. For instance, we analyze the roles of heightened public awareness, evolving patient expectations, and the expansion of legal services following the reform, providing a clearer understanding of these dynamics.

6. **Overstatement of Conclusions**

We appreciate your caution regarding attributing judicial inefficiencies solely to the reform. The conclusions have been revised to adopt a more balanced perspective, acknowledging the multifactorial nature of these inefficiencies and their broader systemic context.

7. **Actionable Recommendations**

The recommendations section has been revised to include greater specificity. For example, we propose practical steps for implementing alternative dispute resolution mechanisms, such as piloting mediation programs and offering specialized training for legal professionals. Additionally, we suggest strategies to expand judicial capacity, including increasing the number of judges and investing in court infrastructure.

8. **Language and Grammar**

The manuscript has undergone thorough review, including careful proofreading and revisions by both authors, as well as additional checks for typographic, grammatical, and language issues. We are confident that these efforts have significantly enhanced the clarity and readability of the text.

By addressing these points, we have sought to improve the manuscript’s methodological rigor, contextual depth, and practical relevance. We deeply appreciate your thoughtful feedback, which has significantly contributed to enhancing the quality and impact of our research.
