# Supplementary material for: Legal education reform and medical litigation: Improved access but delayed justice in plastic surgery malpractice cases in South Korea
Source: PLoS One. 2025 May 29;20(5):e0323059. doi: 10.1371/journal.pone.0323059 (PMC12121745; doi:10.1371/journal.pone.0323059)
Supplement: Attachment — Submitted filename: renamed_bce76.docx [file pone.0323059.s004.docx]

**Comments/ Observations on Manuscript titled:**

**Legal Education Reform and Medical Litigation: Improved Access**

**but Delayed Justice in Plastic Surgery Malpractice Cases in South**

**Korea (Manuscript Number: PONE-D-24-54910)**

The following are the comments/observations:

**Comment 1:** The abstract should capture methodology of the study.

**Response:** It is done and okay.

**Comment 2:** The introduction is not so explicitly elaborated to elicit various variables of the study.

**Response:** It has been addressed as suggested.

**Comment 3:** It is important to elucidate on the necessity of legal education reform in South-Korea, and explain the conceptualization of “more lawyers”, “more lawsuits” and “longer delays”.

**Response:** The title has been modified and the content is aligned with the title of the manuscript.

**Comment 4:** The introduction should explain the rationale for the study, the gap to be filled and the novelty of the study.

**Response:** It has been done as suggested.

**Comment 5:** More literature review should be added to introduction since there is no subheading for literature review part. The following should be considered regarding the review of more studies:

i. Explain a transition from a largely apprenticeship-based system to a more structured graduate-level education model in South-Korea.
ii. Concerns about quality which arose about inconsistencies in the quality of legal education and preparedness of graduates from the realities of legal practices should be elaborated in details.
iii. Standardized curriculum which reform was introduced covering core legal subjects should be explicated.
iv. Explain competitive entrance examination (LEET) which was introduced to select candidate for law school based on aptitude and academic achievement.
v. Explain current challenges and future direction.

**Response:** It All the abovementioned have been explained in the content as pointed out.

**Comment 6:** Elaborate on how leveraging technology into legal education can enhance efficiency and learning outcomes in South-Korea as in the cases of UK and Singapore.

**Response:** This part is not addressed as suggested and no justifiable reason is given for not addressing it.

**Comment 7:** The researcher (s) should explore legal education reform from some selected countries such as: US, UK, Canada through legal education reform in South-Korea can learn from different lessons.

**Response:** This has addressed in the content in p. 38.

**Comment 8:** Regarding ethical considerations, the researcher (s) posited that: “This study was conducted in compliance with ethical standards for research”. Explain what you mean by ethical standards for research and justify with literature.

**Response:** It has been addressed as suggested.

**Comment 9:** Under “Data Analysis”, the researcher should write the full meaning of SPSS as shown the paper.

**Response:** It has been addressed in p. 16 as suggested.

**Comment 10:** Tables1 to 4 should be incorporated into the appropriate places in the contents of the paper.

**Response:** It has been done.

**Comment 11:** The organization and arrangement of the paper should align with the standard of the journal.

**Response:** This should be check by the editor or editor in chief.

**Comment 12:** The results should be elaborated using various studies to justify the overall findings of the study.

**Response:** It has been addressed as pointed out.

**Comment 13:** Recheck typographic, spelling and grammatical errors.

**Response:** It has been improved as suggested.
