# Supplementary material for: Legal education reform and medical litigation: Improved access but delayed justice in plastic surgery malpractice cases in South Korea
Source: PLoS One. 2025 May 29;20(5):e0323059. doi: 10.1371/journal.pone.0323059 (PMC12121745; doi:10.1371/journal.pone.0323059)
Supplement: Attachment — Submitted filename: Response to Reviewer v2.docx [file pone.0323059.s005.docx]

**Comment 6: Elaborate on how leveraging technology into legal education can enhance efficiency and learning outcomes in South-Korea as in the cases of UK and Singapore.**

Response: We thank the reviewer for this valuable suggestion. We have added a comprehensive discussion of how the UK and Singapore leverage technology in legal education to enhance efficiency and learning outcomes, particularly in the context of medical litigation training. This addition can be found in the "Legal and International Implications" section. We have described the UK's use of Learning Management Systems and Virtual Reality simulations for medical malpractice proceedings, as well as Singapore's comprehensive approach to digital technology integration through online collaboration tools and AI-powered legal research tools. We have also discussed how these approaches could be applied to address procedural inefficiencies in South Korea's PRSP litigation. These additions are clearly marked in red text in the revised manuscript with track changes, making them easy to identify.
